# Urinary Dickkopf-Related Protein 3—A Potential Long-Term Biomarker for Progressive CKD in Children

**DOI:** 10.1016/j.ekir.2025.02.003

**Published:** 2025-02-07

**Authors:** Katalin Dittrich, Emilia Marczak, Richard Wagner, Wieland Kiess, Lea Maria Merz

**Affiliations:** 1 Division of Pediatric Nephrology, University Children’s Hospital Leipzig, Leipzig, Germany; 2 Department of Pediatric Surgery, University Hospital Leipzig, Leipzig, Germany

**Keywords:** Dickkopf-3, pediatric chronic kidney disease, prognostic urinary biomarker, progression of chronic renal failure

## Introduction

Chronic kidney disease (CKD) is a serious condition that significantly impacts quality of life, morbidity, and mortality. Mortality rates in pediatric patients with CKD are 30 times higher than those in healthy peers. Moreover, children on dialysis face a 20% higher 5-year mortality.^1^^,^^2^ The decline in kidney function is nonlinear, and disease progression shows significant variability.^3^^,^^4^ Despite advancements in risk categorization and prediction models, forecasting CKD progression in children remains challenging, and prognostic markers indicating individual progression are lacking.

Dickkopf-related protein 3 (DKK3) is secreted by stressed tubular epithelial cells, aggravates renal immunosuppressive functions, and ultimately contributes to renal fibrosis.^5^^,^^6^ By examining the urine of approximately 500 individuals with CKD, Zewinger *et al.* showed a significant increase of urinary DKK3 in adult patients with CKD.^7^ Moreover, their findings indicated that DKK3 levels exceeding 4000 pg/mg creatinine (crea) are associated with a subsequent decrease in estimated glomerular filtration rate (eGFR)of 7.6% within the following year.^7^ Recently, Speer *et al.* determined, that pediatric patients with short-term eGFR decrease (within the following 6 months) have significantly higher urinary DKK3 levels.^4^ Here, we aimed to investigate urinary DKK3 as a novel biomarker to predict long-term CKD progression in children. Urinary DKK3 concentrations were measured using an established enzyme-linked immunosorbent assay in 70 patients aged < 18 years with CKD (Supplementary Material).

## Results

### Urinary DKK3 Excretion is Increased at Higher Kidney Disease: Improving Global Outcomes CKD Stages

Our cohort comprised 70 children (30 female, 40 male) aged between 6 months and 18 years with a broad spectrum of kidney diseases (Supplementary Methods, Supplementary Figure S1A and B). To evaluate the potential of DKK3 as a long-term prognostic marker for CKD progression, we monitored kidney function parameters over a median time of 2.7 (range: 1–4.4) years. The change in eGFR (ΔeGFR) per year was calculated for 54 patients based on the difference between eGFR at the time-point of DKK3 measurement in 2019 and the last follow-up.S1

**Figure 1:**
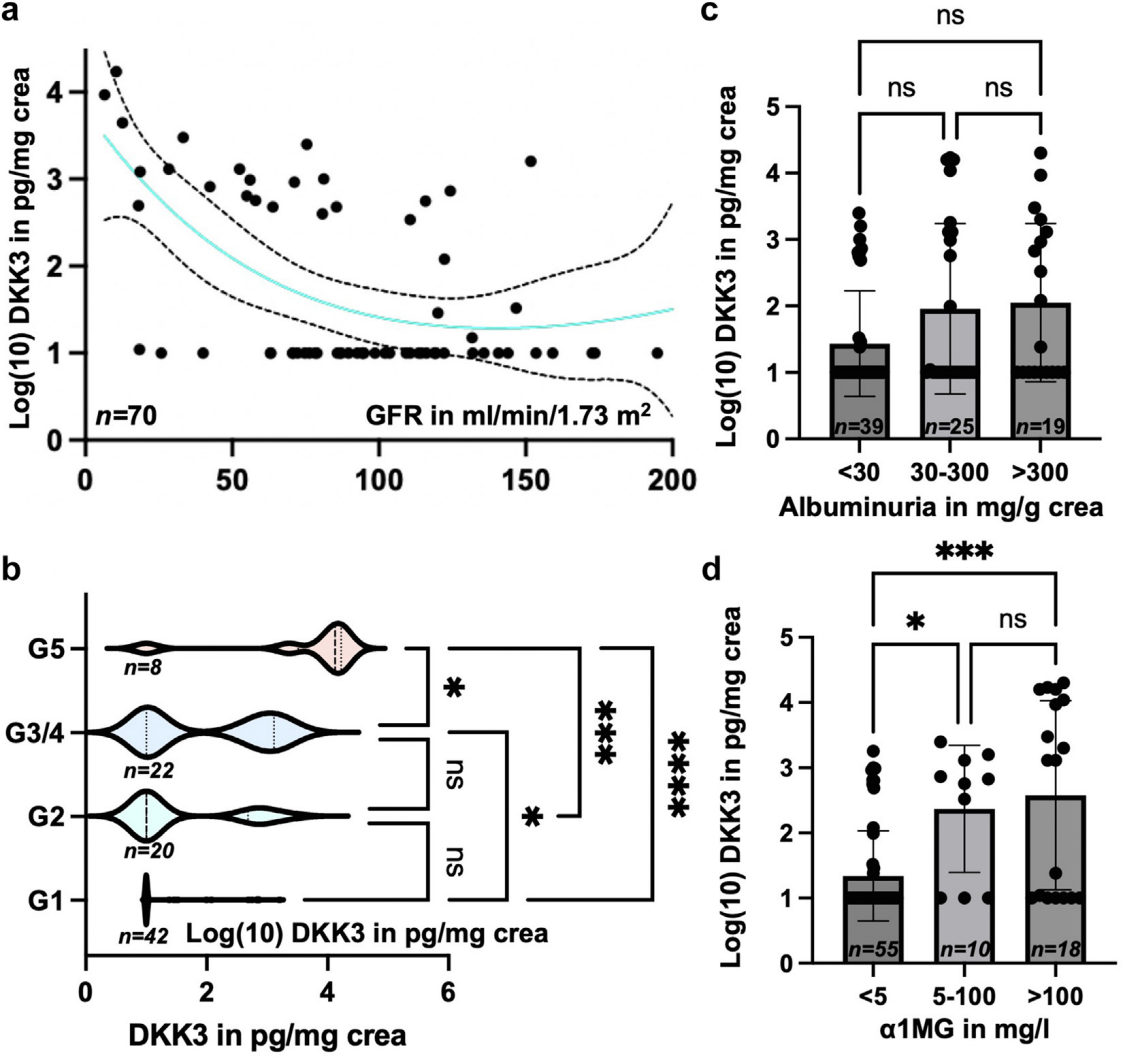
(a and b) Urinary DKK3 inversely correlates with eGFR in children. Nonlinear regression analysis revealed a mild increase of DKK3 values in patients with eGFR values decreasing from 150 to 60 ml/min per 1.73 m^2^. In patients with an eGFR < 60 ml/min per 1.73 m^2^ spline curve showed a steep upward trend in DKK3 values as eGFR increased. Significant difference in urinary DKK3 values between nonadjacent CKD KDIGO groups (Kruskal-Wallis test: G1 and G3/G4: ∗/*P* =0.03, G2 and G5: ∗∗/*P* = 0.002, G1 and G5: ∗∗∗∗/*P* ≤ 0.0001). (c and d) Depicts urinary DKK3 concentrations in pediatric patients with CKD according to albuminuria and α1MG excretion. CKD, chronic kidney disease; DKK3, Dickkopf-related protein 3; eGFR, estimated glomerular filtration rate; KDIGO, Kidney Disease: Improving Global Outcomes; α1MG, α1-microglobulin.

First, we demonstrated that urinary DKK3 and eGFR inversely correlated in children and that DKK3 levels increased with higher CKD Kidney Disease: Improving Global Outcomes stage (Figure 1a and 1b). Low DKK3 concentrations (≤10 pg/mg crea) were frequently detected in patients with an eGFR > 60 ml/min per 1.73 m^2^. Nonlinear regression analysis revealed a shallow increase in DKK3 in patients with eGFR decreasing from 150 to 60 ml/min per 1.73 m^2^. In individuals with an eGFR < 60 ml/min per 1.73 m^2^ we observed a steep increase in DKK3 levels with decreasing eGFR (Figure 1a). Among patients with higher CKD stage (eGFR < 60 ml/min/1.73 m^2^), there were 3 out of 14 who did not secrete DKK3 (“nonsecreters”). We found no significant differences between patient characteristics (nonsecreters vs. secreters) regarding biological sex (male vs. female) albuminuria, α1-microglobulin (α1MG), and disease type (tubular vs. glomerular). Among the patients with increased DKK3 secretion within the CKD stage G1 group (4 of 29), we measured a significantly higher α1MG excretion (Fisher exact test, *P*/∗∗ = 0.0015).

Next, we aimed to investigate whether we could detect significant differences among Kidney Disease: Improving Global Outcomes CKD stages. In patients assigned to Kidney Disease: Improving Global Outcomes CKD stage G1, the median DKK3 concentration was 10 pg/mg crea. For patients in Kidney Disease: Improving Global Outcomes CKD stage G2 and G3, DKK3 predominantly showed 2 clusters (10 pg/mg crea; 630 pg/mg crea). In CKD stage G3/G4, the DKK3 concentration clustered at 1288 pg/mg crea (Figure 1b**)**. Patients in CKD stage G5 had consistently increased concentrations of DKK3, with a median of 15,848 pg/mg crea. Differences between nonadjacent groups were statistically significant (Kruskal-Wallis test: G1 and G3/G4: ∗/*P* = 0.03, G2 and G5: ∗∗/*P* = 0.002, G1 and G5: ∗∗∗∗/*P* ≤ 0.0001) (Figure 1b).

### Urinary DKK3 Concentrations Increase With Higher Urinary α1MG Excretion

Schunk *et al.* described a positive correlation between urinary DKK3 excretion and advancing albuminuria.^8^ In our study, mean DKK3 values increased with higher albuminuria. However, mean DKK3 values in patients with moderate albuminuria (30–300 mg/g crea, mean DKK3 value 2648 mg/g crea) and severe albuminuria (> 300 mg/g crea, mean DKK3 level 1987 mg/g crea) showed similar results and no significant difference in patients without albuminuria (< 30 mg/g crea, mean DKK3 value 219 pg/mg crea) (Figure 1c). DKK3 secretion increased with higher α1MG urine levels, and patients with severe urinary α1MG excretion (>100 mg/l) had significantly higher DKK3 values compared with those without urinary α1MG loss (Figure 1d, Mann-Whitney test: ∗∗∗, *P* < 0.001; ∗,*P* < 0.05).

### Urinary DKK3 Excretion is Increased in Patients With Deteriorating Kidney Function

**Figure 2:**
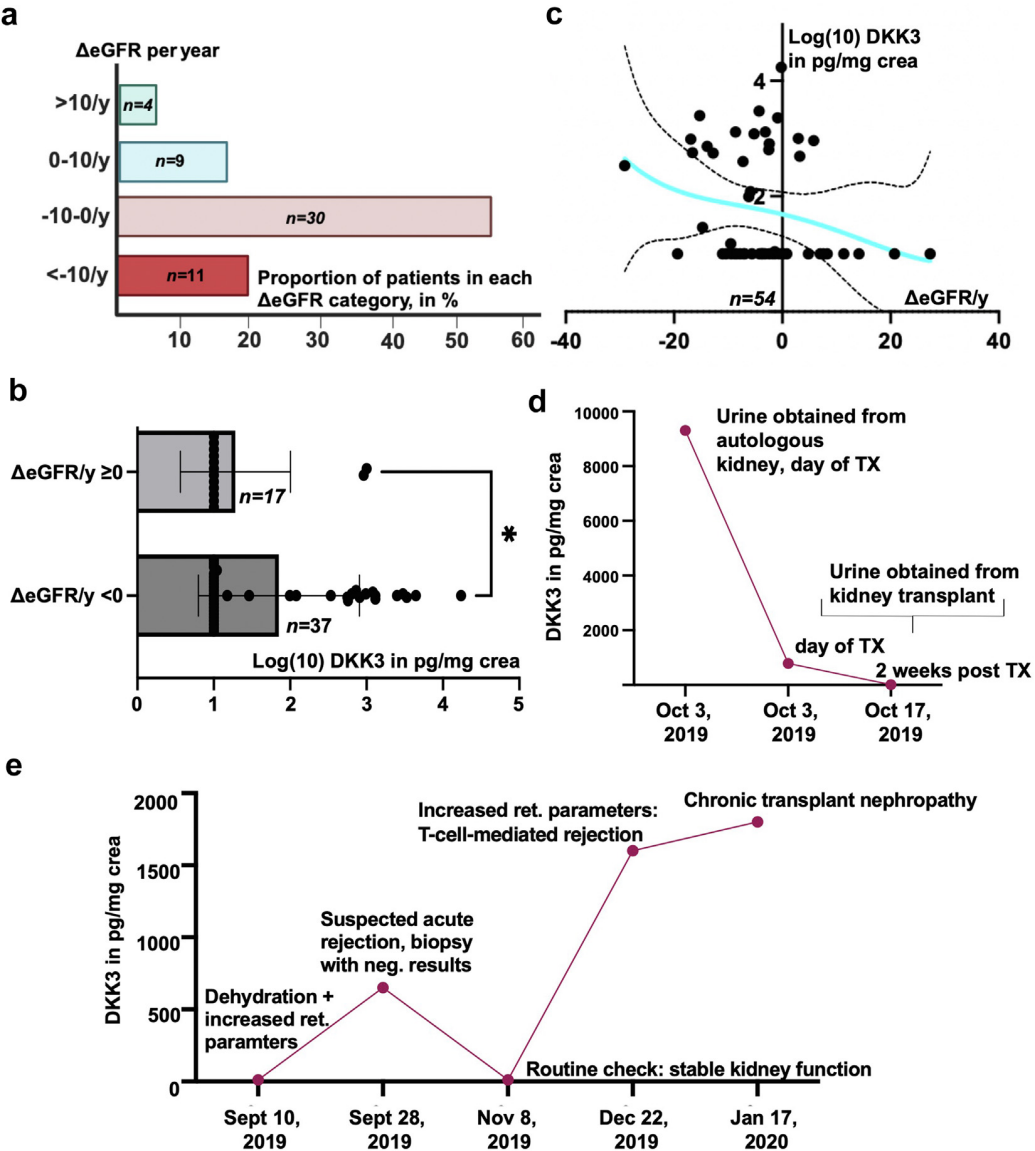
(a) Shows the proportion of kidney function improvement or decline in ΔeGFR per year over a median of 2.7 years. (b) Nonlinear regression showed an increasing urinary DKK3 excretion with decreasing ΔeGFR per year. The maximum detected DKK3 value in a patient with ΔeGFR ≥0 ml/min per 1.73 m^2^ per year was 1000 pg/mg crea. (c) Significant difference in ΔeGFR of patients experiencing improving kidney function compared with those with eGFR decline. (d and e) Individual course of urinary DKK3 excretion in pediatric patients pretransplant and posttransplant. Both patients underwent preemptive kidney transplantation because of renal dysplasia associated with CAKUT. In Patient D, urine from native kidneys and the transplanted kidney was obtained separately (using a vesicostomy and a ureterostomy), whereas in Patient E, "mixed" urine from the bladder was serially examined. CAKUT, congenital abnormalities of the kidneys and urinary tracts; DKK3, Dickkopf-related protein 3; ΔeGFR, change in estimated glomerular filtration rate.

Next, we determined ΔeGFR per year in 54 pediatric patients after a median follow-up duration of 2.7 years. Of these patients, 76% developed a kidney function decline with 56% showing a moderate (ΔeGFR of 0–10 ml/min per 1.73 m^2^ per year) and 20% exhibiting a severe eGFR loss (ΔeGFR >10 ml/min per 1.73m^2^ per year). In addition, 24% were able to maintain or improve kidney function (ΔeGFR ≥ 0 ml/min per 1.73 m^2^ per year, Figure 2a**)**. By comparing patients with kidney function deterioration (ΔeGFR ≤ 0 ml/min per 1.73 m^2^ per year) with those who maintained or improved kidney function (ΔeGFR ≥ 0 ml/min per 1.73 m^2^ per year), we observed a significant difference in urinary DKK3 values (∗, *P* = 0.036), Figure 2b and c). After adjusting for confounding variables, we found that higher urinary DKK3 (*P* = 0.05) and severe mixed kidney disease (*P* = 0.06) were inversely linked to ΔeGFR (Figure 2c, Supplementary Table S1). The mean urinary DKK3 concentration was 193 pg/mg crea in patients with stable or improving kidney function, compared with 912 pg/mg crea in children with deteriorating kidney function. The highest DKK3 value measured in a patient with positive ΔeGFR per year was 1000 pg/mg crea, and values surpassing 1000 pg/mg crea were consistently correlated with kidney function decline. To demonstrate individual trajectories of DKK3 excretion, we performed multiple measurements in 2 patients with end-stage kidney disease who received a kidney transplant. Notably, we observed a normalization of urinary DKK3 secretion within 2 weeks posttransplant, and DKK3 values correlated with transplant condition (Figure 2d and e). We compared patients with primary tubular, glomerular, or mixed renal diseases and did not detect significant differences in urinary DKK3 levels (Supplementary Figure S1C).

## Discussion

DKK3 has emerged as a promising short-term marker for CKD progression in adult and pediatric patients.^4^^,^^7^^,^^9^ We studied urinary DKK3 as a novel prognostic marker for long-term CKD progression in children. We determined an inverse correlation between urinary DKK3 excretion and eGFR, alongside significant variations in urinary DKK3 levels among patients with or without severe α1MG secretion. The strong correlation between increased DKK3 and α1MG excretion, along with the higher α1MG secretion in patients with CKD stage G1 showing elevated DKK3, may be due to DKK3’s origin from tubular cells. Our data show a significant difference in ΔeGFR per year between patients with declining kidney function and those showing improvement, supporting DKK3 as a potential long-term biomarker for CKD. However, urinary DKK3 was exclusively measured at the beginning of the study, which represents a limitation of this study. Children with improving kidney function maintained < 1000 pg/mg crea, suggesting that closer monitoring is needed for those exceeding this threshold.

In adults, urinary DKK3 secretion can be associated with comorbidities such as diabetes or hypertension, whereas pediatric CKD progression seems to be primarily driven by the intrinsic activity of the underlying disease and transplant function.^4^ Consequently, DKK3 levels can serve as a parameter for monitoring individual disease trajectory, especially in kidney transplant recipients. Interestingly, the increase in DKK3 levels was dependent on the underlying cause of kidney function deterioration, showing relevant changes during rejection episodes and chronic transplant nephropathy.

To the best of our knowledge, this is the first study to propose urinary DKK3 as a potential long-term prognostic marker for progressive CKD in children. Although further validation is required, urinary DKK3, alongside eGFR or albuminuria measurements might in the future improve CKD prediction and monitoring of kidney transplant function.

## Disclosure

All the authors declared no conflicting interests.

## Data Availabilty Statement

This study involves a retrospective analysis of urinary DKK3 levels and associated patient data. Patient data are not publicly available, and no public databases were used. Access to the data is restricted to protect patient privacy. Data sharing is not applicable for this study because the datasets generated and analyzed during the study are not publicly accessible. However, specific data requests can be considered on a case-by-case basis, subject to institutional review board approval and the necessary confidentiality agreements.

## Supplementary Material

Supplementary File (PDF and JPEG) **Supplementary Methods. Supplementary References. Figure S1.** (A and B) Shows distribution of diseases causing CKD, age and 117 sex distribution within the pediatric cohort. (C) Indicates no significant difference in 118 urinary DKK3 excretion based on the disease type. **Table S1.** Results of Multiple Linear Regression Analysis of ΔeGFR.

## Footnotes

Supplementary File (PDF and JPEG)

**Supplementary Methods.**

**Supplementary References.**
**Figure S1.** (A and B) Shows distribution of diseases causing CKD, age and 117 sex distribution within the pediatric cohort. (C) Indicates no significant difference in 118 urinary DKK3 excretion based on the disease type. **Table S1.** Results of Multiple Linear Regression Analysis of ΔeGFR.
