# Supplementary material for: Urinary Dickkopf-Related Protein 3—A Potential Long-Term Biomarker for Progressive CKD in Children
Source: Kidney Int Rep. 2025 Feb 7;10(5):1582–6. doi: 10.1016/j.ekir.2025.02.003 (PMC12142503; doi:10.1016/j.ekir.2025.02.003)
Supplement: Supplementary File (PDF and JPEG) — Supplementary Methods. Supplementary References. Figure S1. (A and B) Shows distribution of diseases causing CKD, age and 117 sex distribution within the pediatric cohort. (C) Indicates no significant difference in 118 urinary DKK3 excretion based on the disease type. Table S1. Results of Multiple Linear Regression Analysis of ΔeGFR. [file mmc1.pdf]

Supplementary methods

Study Population

We aimed to investigate urinary DKK3 as novel biomarker to predict long-term CKD progression in children. We therefore collected urine samples from 70 patients with CKD. Predominant conditions were congenital malformations of the kidney and urinary tract (CAKUT), accounting for 38.6% (**Suppl. Fig. 1A**). The second most frequent cause of CKD was glomerular diseases (24.3%), followed by polycystic kidney disease (8.6%) and systemic genetic diseases with kidney involvement, such as Tuberous Sclerosis Complex, Neurofibromatosis Type 1 and Cystinosis (7.1%). Nephronophthisis and tubulopathies were diagnosed in 5.7% and 8.6% of the enrolled cases, respectively (**Suppl. Fig. 1A**). The category "other etiologies" encompassed a broad spectrum of CKD causes, including renal vein thrombosis, lupus nephritis, CKD following acute kidney injury and CKD of unknown origin. The cohort included participants ranging from infancy to adolescence, with a fairly even distribution between ages 1 and 12 (**Suppl. Fig. 1B**). Infants under 1 year represented the smallest group (8.6%), while the largest group comprised adolescents aged 12–18 years (31.4%, **Suppl. Fig. 1B**). Male participants accounted for 57.1%, and females for 42.9% (**Suppl. Fig. 1B).**

Laboratory Analyses

The study cohort consisted of patients exclusively recruited from the Pediatric University Hospital Leipzig, including both inpatients and nephrology outpatient attendees. Urinary DKK3 concentrations were measured in spot urine samples collected at study inclusion in 2019. Standard laboratory analyses, including cystatin C, serum creatinine, urea levels, and albuminuria in spot urine, were performed at baseline (2019) and during follow-up visits through 2023. Follow-up evaluations, involving the collection of urine samples and eGFR measurements, were successfully completed in 54 out of 70 patients. eGFR was calculated using the ped(z) calculator, which applies both the Grubb's equation (2014) and the Schwartz bedside formula (2009)[S1].

Urinary DKK3 Measurement

Spot urine was obtained from participants, immediately frozen at -80°C and urinary DKK3 measurement was performed by the Limbach Group in Heidelberg, Germany. The detailed methodology for measuring DKK3 in urine samples has been published[2–

[4], in brief: The German cancer research center in Heidelberg, Germany identified DKK3 antibodies for a novel ELISA specialized on DKK3 detection and certified for diagnostic use in humans within the European Union (**ReFiNE; DiaRen UG, Homburg/Saar, Germany**). Samples and standards were pipetted and analyzed, using the Tecan Freedom EVO 200 instrument and the Siemens BEP III Analyzer. Absorption was measured at 450 nm by a microplate spectrophotometer. DKK3 concentrations were determined based on the absorption readings of the samples, and the standard curve was generated using six standards provided in the test kit (BESX Software by Siemens). Urinary DKK3 levels were quantified after excluding cross reactivity with other DKK family proteins (i.e., DKK1 and DKK2), normalized to urinary creatinine concentrations to adjust for urine dilution and reported as DKK3-to-creatinine ratio (pg/mg).

Statistical Analysis
Statistical analyses were conducted using GraphPad Prism version 10.1. DKK3 values were logarithmically transformed, and significant differences were assessed using Kruskal-Wallis and Mann-Whitney tests. A p-value <0.05 was considered statistically significant. To illustrate the association between urinary DKK3 excretion and eGFR or ΔeGFR/y a fitted curve using a fourth-order polynomial nonlinear regression model, including 95% confidence intervals, was used. To account for the association between higher urinary DKK3 levels and declining eGFR during follow-up, we controlled for potential confounding variables using multiple linear regression analysis. The analysis was performed in GraphPad Prism, incorporating the following variables: age, sex, urinary DKK3, albuminuria, urinary α1-microglobulin (α1m), initial KDIGO stage, and disease type (tubular, glomerular, or mixed).

Supplementary Table 1: Results of Multiple Linear Regression Analysis of ΔeGFR

Parameter estimates	Variable	Estimate	Standard error	95% CI	P value	P value summary
β0	Intercept	18.69	8.842	0.7604-36.63	0.04	*
β1	DKK3 Concentration	-4.105	2.043	-8.247-0.0382	0.05	*
β2	Disease type<br>1: tubular<br>2: glomerular<br>3: mixed[1]	-4.242	3.731	-11.81 – 3.324	0.3	ns
β3	Disease type<br>1: tubular	-8.397	4.297	-17.11 -0.318	0.06	Borderline significant

	2: glomerular<br>3: mixed [3]					
β4	männlich?[1]	-0.6290	3.008	-6.729 – 5.471	0.8	ns
β5	Age in years	-0.5195	0.2956	-1.11- 0.0799	0.1	ns
β6	GFR rev. Sch warz Formel	-0.05651	0.08278	-0.224<br>– 0.1114	0.5	ns
B7	Albumin<br>1: 30, 2: 30-300, 3: >300 [1]	-1.139	4.311	-9.88 – 7.604	0.8	ns
B8	Albumin<br>1: 30, 2: 30-300, 3: >300 [2]	-2.864	4.643	-12.28 – 6.553	0.5	ns
B9	A1MG<br>1 <5<br>2 5-100<br>3>100[1]	4.905	4.190	-3.593 – 13.40	0.3	ns
B10	A1MG<br>1 <5<br>2 5-100<br>3>100[3]	6.060	7.192	-8.527 – 20.65	0.4	ns

Analysis Plan

This study evaluates urinary DKK3 as a biomarker for predicting long-term kidney function decline in pediatric CKD patients. Secondary objectives include assessing the correlation between urinary DKK3 and eGFR, examining differences by proteinuria severity, and conducting a pilot analysis on the relationship between urinary DKK3 and GFR deterioration.

Study Cohort and Measurements

Cohort: 70 pediatric CKD patients (6 months–18 years) from Pediatric University Hospital Leipzig.

Baseline Measurements:

Urinary DKK3: Measured via certified ELISA in spot urine samples, normalized to creatinine.

eGFR: Calculated with the Grubb's equation and Schwartz formula using the ped(z) calculator.

Proteinuria/Albuminuria: Measured via albuminuria and urinary α1-microglobulin.

Blood Parameters: Serum creatinine, cystatin C, and urea levels.

Follow-Up: Median follow-up of 2.7 years; repeat eGFR and urine measurements in 54 patients.

Statistical Analysis

Urinary DKK3 and eGFR Correlation: Model inverse relationships using a fourth-order polynomial nonlinear regression model**.**

Proteinuria-Based Differences: Compare DKK3 levels across proteinuria severity groups.

Prognostic Analysis:

- Assess ΔeGFR trajectories (categorized as improved, moderate decline, or severe decline).
- Compare urinary DKK3 concentrations between ΔeGFR trajectory groups.

Methods:

- Log-transform DKK3 values to normalize the distribution.
- Adjust results for potential confounding variables by performing multiple linear regression analysis of ΔeGFR. The following variables were included: age, sex, urinary DKK3, albuminuria, urinary α1-microglobulin (α1m), initial KDIGO stage, and disease type (categorized as tubular, glomerular, or mixed).
- Group comparisons will be conducted using Kruskal-Wallis and Mann-Whitney U tests.

Scientific Relevance

This pilot study highlights urinary DKK3's potential as a biomarker for CKD progression, demonstrating significant inverse correlations with advancing disease stages and ΔeGFR. Notably, urinary DKK3 levels consistently exceeding 1000 pg/mg creatinine are strongly linked to kidney function decline in children.

These findings lay the foundation for larger studies incorporating longitudinal DKK3 measurements and additional biomarkers to enhance CKD monitoring and risk stratification in pediatric patients.

Supplementary References

S1. Ped(z), pediatric eGFR calculator. https://www.pedz.de/de/gfr.html

Supplementary Figure 1: A-B) Shows distribution of diseases causing CKD, age and sex distribution within the pediatric cohort. **C)** Indicates no significant difference in urinary DKK3 excretion based on the disease type.

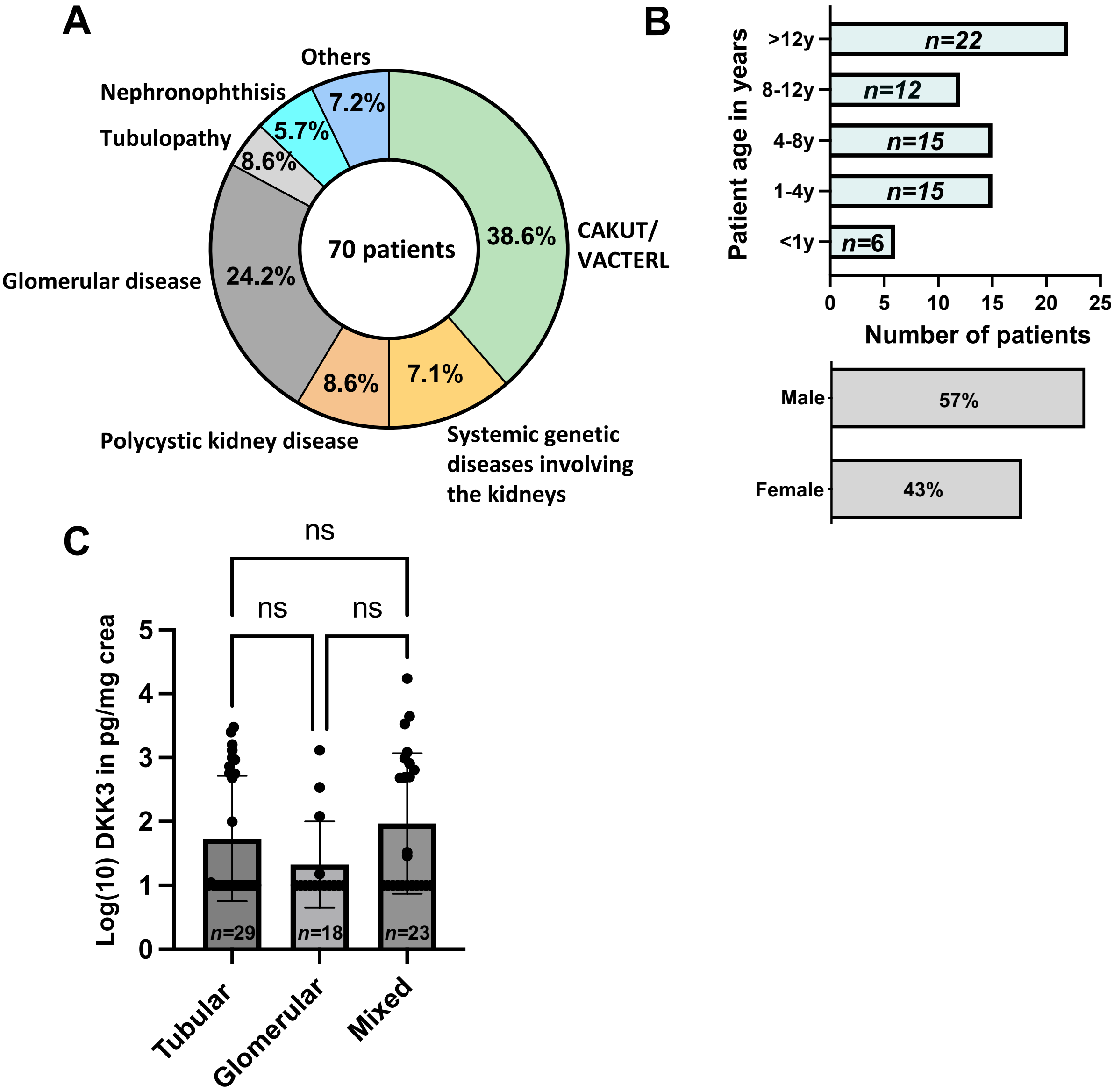

A
Others
Nephronophthisis
Tubulopathy
7.2%
5.7%
8.6%
Glomerular disease
24.2%
70 patients
38.6%
CAKUT/
VACTERL
8.6%
7.1%
Polycystic kidney disease
Systemic genetic
diseases involving
the kidneys
B
Patient age in years
>12y
8-12y
4-8y
1-4y
<1y
n=22
n=12
n=15
n=15
n=6
0
5
10
15
20
25
Number of patients
Male
57%
Female
43%
C
ns
ns
ns
Log(10) DKK3 in pg/mg crea
5
4
3
2
1
0
n=29
n=18
n=23
Tubular
Glomerular
Mixed
